# Multipronged Attack of Stem Cell Therapy in Treating the Neurological and Neuropsychiatric Symptoms of Epilepsy

**DOI:** 10.3389/fphar.2021.596287

**Published:** 2021-03-17

**Authors:** Nadia Sadanandan, Madeline Saft, Bella Gonzales-Portillo, Cesar V. Borlongan

**Affiliations:** Department of Neurosurgery and Brain Repair, University of South Florida Morsani College of Medicine, Tampa, FL, United States

**Keywords:** neuropharmacology, neural circuits, epilepsy, psychiatric comorbidities, complementary and alternative therapies, stem cells

## Abstract

Epilepsy stands as a life-threatening disease that is characterized by unprovoked seizures. However, an important characteristic of epilepsy that needs to be examined is the neuropsychiatric aspect. Epileptic patients endure aggression, depression, and other psychiatric illnesses. Therapies for epilepsy can be divided into two categories: antiepileptic medications and surgical resection. Antiepileptic drugs are used to attenuate heightened neuronal firing and to lessen seizure frequency. Alternatively, surgery can also be conducted to physically cut out the area of the brain that is assumed to be the root cause for the anomalous firing that triggers seizures. While both treatments serve as viable approaches that aim to regulate seizures and ameliorate the neurological detriments spurred by epilepsy, they do not serve to directly counteract epilepsy’s neuropsychiatric traits. To address this concern, a potential new treatment involves the use of stem cells. Stem cell therapy has been employed in experimental models of neurological maladies, such as Parkinson’s disease, and neuropsychiatric illnesses like depression. Cell-based treatments for epilepsy utilizing stem cells such as neural stem cells (NSCs), mesenchymal stem cells (MSCs), and interneuron grafts have been explored in preclinical and clinical settings, highlighting both the acute and chronic stages of epilepsy. However, it is difficult to create an animal model to capitalize on all the components of epilepsy due to the challenges in delineating the neuropsychiatric aspect. Therefore, further preclinical investigation into the safety and efficacy of stem cell therapy in addressing both the neurological and the neuropsychiatric components of epilepsy is warranted in order to optimize cell dosage, delivery, and timing of cell transplantation.

## Introduction

Epilepsy is a neurological illness that features multiple unprovoked seizures as a result of aberrant cerebral activity (Hauser and Kurland, 1975; Fisher et al., 2005; Fisher et al., 2014). Across the world, sixty million people suffer from epilepsy, with approximately 2.3 million afflicted in the United States (Jobst and Cascino, 2015; “comorbidity,” 2016). Temporal lobe epilepsy (TLE), which occurs in 30% of epileptic patients, manifests in intricate partial seizures, hippocampal limbic deterioration, and concomitant neuropsychiatric maladies (Devinsky, 2004; Lewis, 2005; Shetty and Upadhya, 2016). The International League Against Epilepsy established two categories of seizures: generalized seizures and focal seizures. Generalized seizures are bilateral, transpire swiftly and emanate from one site and are divided into these subclasses: tonic-clonic, absence, clonic, tonic, myoclonic, and atonic. Stemming from one hemisphere, focal seizures can remain in the initial epileptic region or move to other locations (Berg et al., 2010). Diagnosis of epilepsy falls into three major categories: structural or metabolic, genetic, and unknown source. Genetic epilepsy is generated directly by a genetic mutation that induces seizures. Structural/metabolic epilepsy is ultimately engendered by either a structural lesion or metabolic illness. Seizures spurred by injuries, such as stroke or infection, are instances of structural/metabolic epilepsy. An unknown cause epilepsy can be due to a genetic variable or a separate disorder, but the root cause behind these seizures is unknown (Berg et al., 2010). Epilepsy also displays cognitive and behavioural impairments, which resemble the symptoms that accompany neuropsychiatric illnesses. These symptoms include hallucination, delusion, apathy, altered cognition, delirium, etc. (Rektor et al., 2015; Miyoshi et al., 2016). Other psychiatric illnesses that commonly arise with epilepsy include anxiety, depression, and obsessive-compulsive disorder (Verrotti et al., 2014; Kanner, 2016a).

**FIGURE 1:**
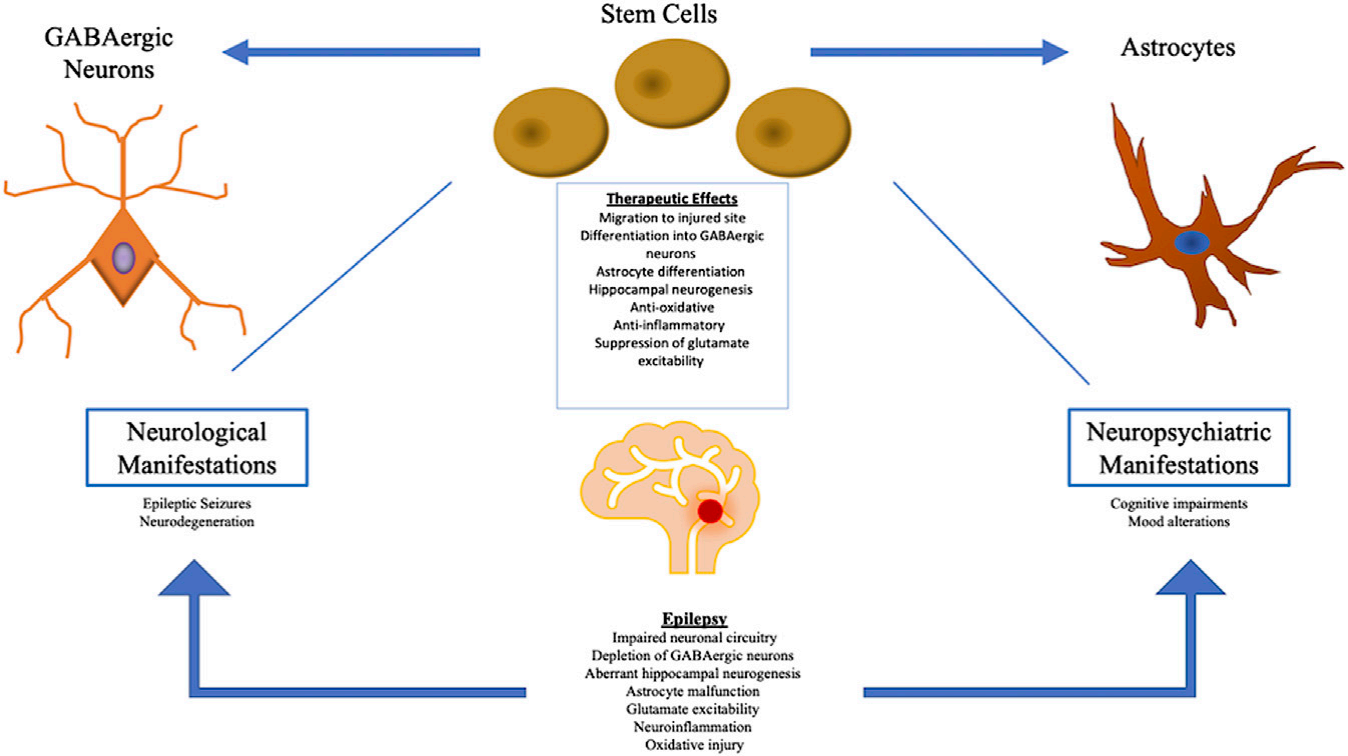
The pathophysiology of epilepsy manifests in both neurological and neuropsychiatric symptoms. Stem cell therapy addresses both sets of symptoms by inducing a multitude of therapeutic mechanisms, primarily through the restoration of GABAergic depletion and production of healthy astrocytes at the injured site. Stem cells also promote normal hippocampal neurogenesis and attenuate oxidative/inflammatory activity, as well as glutamate excitability.

Currently, the main treatments available for epilepsy include pharmacological medications and surgery. Antiepileptic drugs, AEDs, are the major source of treatment among epileptic patients (Stafstrom and Carmant, 2015). AEDs aim to block sodium ion and other cation protein channels, thereby attenuating firing in the brain. AEDs also work by stimulating gamma-Aminobutyric acid (GABA) channels (Stafstrom and Carmant, 2015). When AED drugs are ineffective, surgical resection of the temporal lobe serves as another potential therapy. Surgery involves removing portions of the cerebral tissue that induce seizures (Ramey et al., 2013). Interestingly, AEDs address the neuropsychiatric symptoms indirectly via the suppression of heightened neuronal firing and blockage of present pathways (Rektor et al., 2015). Epileptic treatment utilizing deep brain stimulation modifies neuronal firing in limbic areas and therefore, also indirectly ameliorates neuropsychiatric symptoms (Shetty and Upadhya, 2016). Nonetheless, further investigation needs to be conducted in order to elucidate therapies that address both the neurological and neuropsychiatric aspects of epilepsy (Shetty and Upadhya, 2016). Recently, autologous stem cell transplantation has become a potential treatment for epilepsy which addresses both neurological and neuropsychiatric effects. Stem cell therapy has been explored for the treatment of several neurological diseases including multiple sclerosis, stroke, and Parkinson’s disease (Chu et al., 2004). Having been employed in a wide-range of neurological and neuropsychiatric maladies, stem cell therapy bears significant therapeutic potential in epilepsy, aiming to bolster stem cell differentiation into GABAergic neurons and amplify the GABAergic lineage, which in turn, helps ameliorate the GABA-deficient neural firing pathways observed in epilepsy (Shetty and Upadhya, 2016). As depicted in Table 1, preclinical and clinical investigations have explored various cell types for the treatment of epilepsy, such as hippocampal precursor cells, NSCs, GABAergic precursor cells, and systematic delivery of bone marrow-derived mononuclear cells and mesenchymal cells (Scheibel et al., 1974; Rao et al., 2006; Waldau et al., 2010; Shetty and Upadhya, 2016; Papazian et al., 2018; Xu et al., 2019; Xian et al., 2019). NSCs, MSCs and interneuron precursors in epilepsy work to improve neuronal circuitry and restore GABAergic neurons. Furthermore, the neurological and neuropsychiatric aspects of epilepsy are amenable to therapeutic mechanisms of stem cells, as shown in Figure 1 (Valenzuela et al., 2007; Braun and Jessberger, 2014). These stem cell treatments have been explored through clinical and preclinical trials; however further research needs to be conducted to investigate the efficacy of these stem cell transplantations. This review explores the safety and efficacy of stem cell therapy as a novel treatment for epilepsy, and investigates the potential mechanisms underlying stem cells’ therapeutic potency against the neurological and neuropsychiatric components of epilepsy.

**TABLE 1:** Recent advances supporting the therapeutic use of stem cells in epileptic treatment.

Type of study (Preclinical/Clinical)	Title, Author, Year	Stem cell variety	Significant findings
Preclinical	*Mesenchymal Stem Cell Protection of Neurons against Glutamate Excitotoxicity Involves Reduction of NMDA-Triggered Calcium Responses and Surface GluR1, and Is Partly Mediated by TNF*, Papazian et al. (2018)	MSCs	*In vitro* and *in vivo*, MSC administration correlated with downregulation of NMDAR expression and glutamate-induced calcium ion activity, indicating that MSCs can shield neurons from glutamate excitotoxicity spurred by epilepsy (Papazian et al., 2018)
Preclinical	*Intravenous infusion of mesenchymal stem cells reduces epileptogenesis in a rat model of status epilepticus,* Fukumura et al. (2018)	MSCs	Systematically infused MSCs migrated to the hippocampus of lithium-pilocarpine rats and protected neurons expressing GAD67 and NeuN and resulted in curtailed epileptogenesis and mitigated neurological impairments. Aberrant mossy fiber sprouting in the dentate gyrus also decreased substantially (Fukumura et al., 2018)
Preclinical	*Genome Editing in Neuroepithelial Stem Cells to Generate Human Neurons with High Adenosine-Releasing Capacity,* Poppe et al. (2018)	NESs derived from human embryonic stem cells	NESs designed to lack the ADK gene showed a significant elevation of adenosine production *in vitro*, indicating that these genetically engineered NESs may serve as useful adenosine carriers, mitigating abnormal adenosine homeostasis in epilepsy (Poppe et al., 2018)
Preclinical	*Neurochemical properties of neurospheres infusion in experimental-induced seizures*, de Gois da Silva et al. (2018)	NSCs	In pilocarpine, pentylenetetrazole, and picrotoxin rats, NSC intravenous delivery generated antioxidant effects, lowering levels of glutathione, superoxide dismutase and catalase (de Gois da Silva et al., 2018)
Preclinical	*Human induced pluripotent stem cell-derived MGE cell grafting after status epilepticus attenuates chronic epilepsy and comorbidities via synaptic integration*, Upadhya et al. (2019)	hiPSC-derived MEG-like precursor cells	In a SE model, the transplanted cells migrated swiftly to the hippocampus and differentiated into fully developed inhibitory interneurons, as well as secreting a wide range of therapeutic neuropeptides. The precursor transplantation resulted in inhibited SRS and improved cognitive, mood, and memory deficits. In addition, interneuron deterioration, abnormal moss fiber sprouting in the dentate gyrus, and anomalous neurogenesis were mitigated (Upadhya et al., 2019)
Preclinical	*Human Pluripotent Stem Cell-Derived Striatal Interneurons: Differentiation and Maturation In Vitro and in the Rat Brain*, Noakes et al. (2019)	hPSC-derived MGE/CGE like progenitor cells	hPSC-derived MGE/CGE like progenitor cells differentiated into fully developed striatal interneurons *in vitro* and following transplantation into neonatal rats, the progenitor cells evolved into subclasses of striatal interneurons and migrated to the hippocampus (Noakes et al., 2019)
Preclinical	*Transplanting GABAergic Neurons Differentiated from Neural Stem Cells into Hippocampus Inhibits Seizures and Epileptiform Discharges in Pilocarpine-Induced Temporal Lobe Epilepsy Model*, Xu et al. (2019)	NSCs and NSC-derived GABAergic neurons	Pharmacoresistant epileptic rats received NSCs and NSC-derived GABAergic neuron transplantation in the hippocampus, which culminated in a repressed frequency of electroencephalography GABAergic neurons demonstrated substantial homing capacity, moving to the injured hippocampus more swiftly than NSCs (Xu et al., 2019)
Preclinical	*Mesenchymal stem cell-derived exosomes as a nanotherapeutic agent for amelioration of inflammation-induced astrocyte alterations in mice*, Xian et al. (2019)	MSCs	MSC-Exo administration to LPS-conditioned hippocampal astrocytes attenuated epilepsy-induced alterations in astrocytes, as astrogliosis and neuroinflammation decreased in SE mice following exosomal treatment. The SE mice exhibited ameilorated cognitive impairments, showing improvements in learning and memory (Xian et al., 2019)
Preclinical	*Intranasally Administered Human MSC-Derived Extracellular Vesicles Pervasively Incorporate into Neurons and Microglia in both Intact and Status Epilepticus Injured Forebrain*, Kodali et al. (2019)	hMSCs	Intranasal injection of EVs harvested from hMSCs into SE rats resulted in hippocampal neuron incorporation of EVs, primarily in the CA1 sector and entorhinal cortex, which are regions that develop significant neurodegeneration post-epilepsy. Furthermore, hMSCs show potential in ameliorating chronic symptoms of epilepsy by combatting neurodegeneration and imparting neuroprotection (Kodali et al., 2019)
Preclinical	*Human forebrain endothelial cell therapy for psychiatric disorders,* Datta et al. (2020)	Interneuron graft and periventricular endothelial cells	In an experimental model of a psychiatric disease, human periventricular endothelial cells heightened the potency of the interneuron graft by enhancing the migratory abilities of interneurons. As a result, the animal model demonstrated mitigated behavioral impairments (Datta et al., 2020)
Preclinical	*Overexpression of fibroblast growth factor-21 (FGF-21) protects mesenchymal stem cells against caspase-dependent apoptosis induced by oxidative stress and inflammation*, Linares et al. (2020)	BM-MSCs	FGF-21 overexpression in BM-MSCs protected these cells from glutamate excitotoxicity, oxidative stress, and neuroinflammation, which often result from the onset of epilepsy. Genetically modified BM-MSCs displayed inhibited apoptosis, as caspase pathways induced by hydrogen peroxide and TNF-α were suppressed (Linares et al., 2020)
Clinical	*Treatment of refractory epilepsy patients with autologous mesenchymal stem cells reduces seizure frequency: An open label study,* Hlebokazov et al. (2017)	MSCs	Drug-resistant epileptic patients were treated with either AEDs alone or AEDs in conjunction with one dose of autologous MSCs delivered intravenously and a subsequent intrathecal administration of autologous MSCs that had differentiated into neural cells. Over the course of one year, 3 patients in the MSC group out of 10 became seizure free and 5 patients treated with the MSCs became responsive to AEDs, compared to only 2 of 12 patients in the AED group (Hlebokazov et al., 2017)
Clinical	*Multiple Autologous Bone Marrow-Derived CD271+ Mesenchymal Stem Cell Transplantation Overcomes Drug-Resistant Epilepsy in Children,* Milczarek et al. (2018)	BMNCs and BM-MSCs	Drug-resistant epileptic patients received both an intrathecal/intravenous injection dose of BMNCs and an intrathecal dose of BMMSCs. In the two-year follow up, subjects demonstrated improved neurological and cognitive function and mitigated seizure frequency. The stem cell treatment was safe, producing no adverse events (Milczarek et al., 2018)
Clinical	*Safety and seizure control in patients with mesial temporal lobe epilepsy treated with regional superselective intra-arterial injection of autologous bone marrow mononuclear cells*, DaCosta et al. (2018)	BMMCs	Autologous BMMCs were intra-arterially delivered to MTLE patients. Over the course of six months, 40% of the patients displayed seizure remission and cognition significantly improved, as memory scores increased. The transplantation was safe and generated no adverse effects (DaCosta et al., 2018)
Clinical	*Intrathecal Infusion of Autologous Adipose-Derived Regenerative Cells in Autoimmune Refractory Epilepsy: Evaluation of Safety and Efficacy,* Szczepanik et al. (2020)	ADRCs	Autologous ADRCs were delivered intrathecally to autoimmune refractory epileptic patients three times every three months. In the follow-up, patients showed rehabilitated cognitive, social, and motor impairments. While one patient developed seizure remission, other subjects displayed lower degrees of seizure amelioration or no apparent recovery (Szczepanik et al., 2020)

## Epilepsy: A Double-Edged Sword With Neurological and Neuropsychiatric Elements

### The Link Between Epilepsy and Neuropsychiatric Illness

Research indicates that behavioural and psychiatric diseases as well as deficits in social cognition often accompany the neurological symptoms of epilepsy (Kanner, 2008; Mula and Hesdorffer, 2011; Hecimovic et al., 2012; Realmuto et al., 2015). Evidence suggests that the peri-ictal and interictal psychiatric impairments of epilepsy can be linked to psychiatric illnesses (Rektor et al., 2013). Those who suffer from psychiatric perturbations induced by a seizure commonly experience the same disturbances between recurrent seizures (Rektor et al., 2013). Several epileptic patients experience mood alterations, such as depressed moods, and memory deficits (Rektor et al., 2013; Korczyn et al., 2015; Rektor et al., 2015). Other psychological disorders such as bipolar disorder and depression have also been associated with epilepsy (Rektor et al., 2015).

In addition, nocturnal epileptic episodes and AEDs both frequently incite sleep perturbations, making this a potential target for comprehensive therapeutic strategies in epilepsy (Plioplys et al., 2007; Magiorkinis et al., 2010; Rektor et al., 2013; Rektor et al., 2015; Ladino et al., 2016; Mula et al., 2016). Since epilepsy patients often experience impaired sleep, this negatively affects their cognitive function and memory (Walker and Stickgold, 2006). Sleep deprivation may provoke insults in positive and neutral memories, indicating why depressed mood often arises as a psychiatric symptom in epileptic patients (Jensen, 2011; Bullmore and Sporns, 2012; Kramer and Cash, 2012; Rektor et al., 2013). Epilepsy can affect the normal development of neuronal networks in children, making early diagnosis and intervention critical to curtailing the effects of comorbid psychological deficits on the individual (Jensen, 2011; Bullmore and Sporns, 2012; Kramer and Cash, 2012; Rektor et al., 2013). Injuries arising during development may consequently engender cognitive deficits that are often overlooked in epileptic patients and go untreated (Plioplys et al., 2007; Rektor et al., 2015). Further investigation into how epilepsy influences the mind as the mind ages is critical for curating more holistic therapies (Rektor et al., 2013).

As research indicates, epilepsy is concomitant with a multitude of neuropsychiatric maladies. (Kanner and Balabanov, 2002; Kanner, 2006; Hesdorffer and Trimble, 2016). Notably, patients suffering from depression may be more prone to developing seizures than normal individuals. Therefore, a bidirectional correlation among epilepsy and neuropsychiatric illnesses may exist (Hesdorffer et al., 2006; Kanner, 2006). Epileptic patients also tend to experience mood impairments such as depression, anxiety, and personality disorders (Kogeorgos et al., 1982; Hermann et al., 2000; Swinkels et al., 2005; García-Morales et al., 2008). In addition, patients with epilepsy who also display psychiatric disorders exhibit aberrant clinical traits (García-Morales et al., 2008). Psychiatric events can be designated at the inception of epileptic seizures (García-Morales et al., 2008). Epileptic symptoms are divided into the following categories: preictal, ictal, interictal, and postictal (García-Morales et al., 2008). The preictal stage marks the time between the onset of the seizure and up to two days prior (García-Morales et al., 2008). Ictal symptoms can be identified during the seizure (García-Morales et al., 2008). Interictal symptoms manifest independently of the seizure, and the postictal phase begins within five days following the seizure (García-Morales et al., 2008).

Since neuropsychiatric comorbidities often arise in epileptic patients, it is essential for clinicians to take indications of mood impairments into account when treating patients with epilepsy. When conducting a psychiatric assessment of an epileptic patient, it is important to notice symptoms like anhedonia, irritability, decreased self-esteem, reduced concentration, fatigue, etc (Cramer et al., 2003). Many patients also experience anxiety as part of their seizures (Cramer et al., 2003). Almost half of all patients with epilepsy experience postictal anxiety and this can be associated with other mood disorders (Jones et al., 2005). Some epileptic patients display psychosis with inconsistent symptoms. However, the psychotic symptoms demonstrated by epileptic patients are less drastic than nonepileptic patients, and so, diagnosing psychosis in conjunction with epilepsy is complicated (Tadokoro et al., 2007).

Unfortunately, inaccurate diagnosis of psychiatric comorbidities in epileptic patients is common and not only negatively affects the individual, but also generates harmful repercussions in regard to the healthcare system altogether (Lee et al., 2005; Sancho et al., 2008). A late diagnosis negatively impacts the patient, their loved ones, and society (Lee et al., 2005; Sancho et al., 2008). Additionally, evidence has illustrated that the psychiatric component of epilepsy unfavourably influences the effectiveness of surgery and epileptic therapy (Anhoury et al., 2000; Hitiris et al., 2007).

### The Mechanistic Connection Between Epilepsy and Neuropsychiatric Disorders

The physiological foundation of epilepsy and its psychiatric comorbidities require further examination. Indeed, there are some fundamental similarities in the two different diseases that can be observed in animal models for epilepsy (Dailey et al., 1992; Jobe et al., 1999; Ryu et al., 1999). Norepinephrine and serotonin play a central role in the pathophysiology of epilepsy, as observed in experimental models (Dailey et al., 1992; Jobe et al., 1999; Ryu et al., 1999). The depletion of these neurotransmitters exacerbates seizure intensity (Dailey et al., 1992; Jobe et al., 1999; Ryu et al., 1999). However, elevating levels of these neurotransmitters via the administration of reuptake inhibitors leads to a reduction in seizure intensity (Dailey et al., 1992; Jobe et al., 1999; Ryu et al., 1999). Concentrations of neurotransmitters also play a key role in psychiatric disorders (Cheetham et al., 1989). In patients affected with neuropsychiatric disorders, the activity of serotonin receptor (5-HT1A) was analyzed utilizing positron emission tomography (Toczek et al., 2003; Savic et al., 2004; Giovacchini et al., 2005). Reduced 5-HT1A binding was observed in the temporal lobes proximal to the raphe nuclei in both epileptic and mood disorder patients (Toczek et al., 2003; Savic et al., 2004; Giovacchini et al., 2005). Additional investigation into the mechanistic link between epilepsy and neuropsychiatric comorbidities is essential to formulating effective treatment for epilepsy.

## Multimodal Treatments for Epilepsy: Neurological

### AEDs

Presently, treatment of epileptic seizures can be managed by AEDs (Remy and Beck, 2006) that are typically effective in ameliorating symptoms for a large number of epileptic patients (Schmidt and Schachter, 2014). Drug treatments aim to attenuate the intensified electrical activity in the brain by suppressing neuronal firing through the hindrance of sodium channels and calcium channels as well as the glutamate-mediated response (Stafstrom and Carmant, 2015). Another mechanism of action employed by AEDs includes the stimulation of GABAergic secretion and bolstered potassium ion channel conductivity in order to repress neuronal activity (Stafstrom and Carmant, 2015). However, AED resistance can be observed in approximately 30% of patients with epilepsy (Remy and Beck, 2006). While the mechanisms behind pharmacotherapy-resistance are unknown and believed to be spurred by a wide range of genetic and nongenetic determinants, the ABCB1 gene may be involved (Keangpraphun et al., 2015). A crucial transporter that may generate drug efflux is p-glycoprotein that may be coded for by ABCB.1 (Keangpraphun et al., 2015). Patients that suffer from mutations in the ABCB1 gene may develop AED pharmacotherapy-resistance on account of variable expression of the gene, which in turn, hinders AEDs’ mechanism of action (Keangpraphun et al., 2015). Additionally, the alternative forms of the apolipoprotein E in patients correlate with increased risk for some subclasses of epilepsy and may be involved with AED pharmacotherapy-resistance (Gong et al., 2016). Even with the formulation of novel AEDs, the prominence of pharmacotherapy-resistance still remains (Remy and Beck, 2006). Moreover, for treatment of drug-resistant epilepsy, alternative therapeutic approaches, such as surgery, should be explored (López González et al., 2015).

### Surgical Resection

Besides pharmaceutics, other potential therapies for epilepsy involve surgical resection to remove the portion of the brain causing the seizures (López González et al., 2015). Generally, neurosurgery is considered following the inefficacy of around two rounds of AED treatment for pharmacotherapy-resistant patients (López González et al., 2015). Early deliberation for surgery leads to higher quality of life and reduced medical expenses for epileptic patients (López González et al., 2015). In most cases, injury to the temporal lobe occurs with drug-resistant epilepsy and therefore, surgical resection of this impaired region serves as a fruitful therapy that culminates in regulation of seizures and ameliorated neurological impairments (López González et al., 2015). Surgical resection has been shown to be generally safe and effective in epileptic patients, particularly with the epileptic tissue in the temporal lobe (Coyle et al., 2017). Nonetheless, in some cases, surgery may result in exacerbated neurobehavioral complications (Coyle et al., 2017). Amygdalohhippcampectomy serves as another type of surgical resection that has been shown as effective, though it is still invasive (Coyle et al., 2017). Furthermore, while surgical intervention displays significant safety and efficacy against epilepsy, it does carry some limitations and more minimally invasive therapies that more effectively target the neuropsychiatric component of epilepsy should be explored.

### Vagus Nerve Stimulation

Vagus nerve stimulation (VNS) serves as another neuromodulation-related therapy that lessens seizure frequency but does not fully inhibit seizure activity (Englot et al., 2016). A VNS device mirrors a cardiac pacemaker and is placed just inferior to the clavicle, encircling the left vagus nerve (González et al., 2019). While VNS therapy may not culminate in full cessation of seizures in patients, evidence suggests that it does ameliorate quality of life for epileptics (González et al., 2019). A previous investigation has demonstrated that high-frequency VNS lowered the occurrence of seizures in epileptic patients by 25% (González et al., 2019). Along with reducing seizure recurrence, VNS has been shown to mitigate the post-ictal state, mood alterations, learning deficits, and memory impairments (González et al., 2019). While VNS is minimally invasive and is typically safe and effective, VNS therapy has resulted in adverse events in some cases (González et al., 2019), demonstrating the need to investigate additional therapies for epilepsy.

### Ketogenic Diet

Notably, a ketogenic diet has also been explored as a treatment method (Zamani et al., 2016). The ketogenic diet has demonstrated therapeutic potency in both adult and pediatric epilepsy. The ketogenic diet mirrors fasting in that it spurs ketone generation (e.g., β-hydroxybutyrate (BHB), acetoacetate, and acetone). It influences mitochondrial energy metabolism, as it can directly enter the tricarboxylic acid cycle without glycolysis. For epileptic patients who do not show improvement with traditional pharmaceutics and surgical resection, a ketogenic diet can be considered. In a TLE rat model, the ketogenic diet attenuated the frequency of epileptic events via suppression of ADK expression and elevation of adenosine levels (Boison and Steinhäuser, 2018). Evidence indicates that cerebral energy metabolism is substantially elevated following adoption of the ketogenic diet, and neuronal tissue becomes increasingly fortified against stressful conditions. As a consequence, the seizure threshold greatly improves (Boison and Steinhäuser, 2018). With reduced brain glucose metabolism induced by a ketogenic diet, glycolytic ATP may engender the hyperpolarization of neurons. This may attenuate the extent of neuronal excitability, which demonstrates therapeutic benefit in preventing epileptic seizures (Boison and Steinhäuser, 2018).

## Multimodal Treatments for Epilepsy: Neuropsychiatric

### Pharmaceutics

Many patients with epilepsy experience anxiety and depression (Hamilton et al., 2014) and these disorders often go untreated even though they are the most common psychiatric comorbidities for epileptic patients (Selassie et al., 2014). Evidence indicates that psychosocial or neurological deficits may spur the neuropsychiatric components of epilepsy (Cardamone et al., 2013). While anxiety and depression are often overlooked in epilepsy patients, specific, single-item screening procedures could be beneficial in addressing these ailments (Cardamone et al., 2013). Treatment methods for the neuropsychiatric aspect of epilepsy fall into two categories: traditional and non-traditional. Antidepressants, such as selective serotonin reuptake inhibitors (SSRIs) and serotonin-noradrenaline reuptake inhibitors (SNRIs), serve as traditional remedies for neuropsychiatric symptoms (Cardamone et al., 2013). Evidence has demonstrated that antidepressants lessen seizure frequency, but further investigation is necessary to fully elucidate the correlation between antidepressants and epilepsy (Cardamone et al., 2013). While antidepressants can potentially reduce recurrent seizure risk, it is not advisable for physicians to prescribe amoxapine, bupropion, clomipramine, or maprotiline to treat depression in epileptic patients (Górska et al., 2018). However, SSRIs and SNR1s like sertraline and citalopram, display safety and efficacy in treating epilepsy-related depression (Górska et al., 2018) and even bear anticonvulsant activity (Kanner, 2016b; Górska et al., 2018). Indeed, initiating clinical studies to illuminate the connection between antidepressants and epilepsy is a complicated task on account of the long-term follow-up and multifaceted evaluation of results (Cardamone et al., 2013). In a clinical trial investigating the role of antidepressants in epilepsy treatment, seizure frequency was greatly decreased in subjects receiving antidepressants compared to the placebo group (Kanner, 2016b). Nevertheless, overdosing these medications can have dangerous and proconvulsant effects (Kanner, 2016b).

### Non-Traditional Therapies

Non-traditional alternatives to pharmaceutics are currently being examined for epilepsy and include neurostimulation, deep brain stimulation, laser ablation, ultrasound therapy, meditation, and art and musical therapy (Dawit and Crepeau, 2020). The relaxation that ensues from mediation and art/music therapy may not only reduce seizure frequency but may also result in better quality of life by addressing the neuropsychiatric comorbidities of epilepsy (Dawit and Crepeau, 2020). Cognitive behavioural therapy, yoga, and biofeedback have also risen as potential non-invasive therapies for epilepsy (Leeman-Markowski et al., 2017). Fortunately, non-traditional therapies for the neuropsychiatric component of epilepsy are becoming fairly common. In the clinical setting of drug-resistant epilepsy, mindfulness-based therapy demonstrated positive results (Tang et al., 2015). By integrating mindfulness into their lives, patients developed improved quality of life, better mood, and reduced seizure frequency (Tang et al., 2015).

Cannabidiol-based treatments serve as another non-traditional remedy that bears anticonvulsant properties and aids in regulating the neuropsychiatric aspect of epilepsy (Devinsky et al., 2014). The US Food and Drug Administration (FDA) provided approval for cannabidiol (CBD) use in Lennox-Gastaut syndrome (LGS) and Dravet syndrome (DS) (Ryan, 2020). Receptors for G-protein coupled cell membrane cannabinoid type 1 (CB1) are prominent in the cerebral cortex, hippocampus, basal ganglia, and the cerebellum (Ryan, 2020). CB1 has been shown to suppress glutamate secretion into the glutaminergic synapse, indicating its potential to ameliorate both the neurological and neuropsychiatric aspects of epilepsy (Ryan, 2020). Evidently, CBD may suppress heightened excitability of neurons and exert neuroprotection in epilepsy (Ryan, 2020). Nonetheless, CBD treatment can induce adverse side effects, such as drowsiness, diarrhea, vomiting, and reduced appetite (Silva and Del Guerra, 2020). Therefore, evaluating other therapies that can effectively mitigate the neurological and psychiatric aspects of epilepsy is essential.

## The Therapeutic Potential of Stem Cell Therapy in Epilepsy

Stem cell therapy has emerged as a fascinating potential treatment for epilepsy that varies substantially from current treatments. While anticonvulsant medications and electrical stimulation address the symptoms of epilepsy, they do not target the ultimate source. With stem cell therapy, neurotrophic factors are increased, and neurogenesis is promoted. Further investigation is required to evaluate the integrity of NSC grafts and their ability to attenuate SRS and give rise to GABAergic interneurons. The adoption of a regulated procedure to induce stem cell differentiation into needed cell types is key to establishing an effective cell-replacement therapy for epilepsy. Nonetheless, the great differentiative and migratory potential of stem cells indicate risk of tumorigenesis and metastasis. To further elucidate the safety and efficacy of stem cell therapy in epilepsy, the viability of stem cell grafts and length of survival time should be assessed. Indeed, animal models representing status epilepticus (SE) have demonstrated the curative potential of stem cell therapy in epileptic patients. Stem cells have been shown to help alleviate the pathology of epilepsy, impart neuroprotection, diminish the frequency and duration of seizures, and ameliorate memory and learning deficits (Scheibel et al., 1974; Hattiangady et al., 2008; Waldau et al., 2010; Shetty, 2014; Shetty and Upadhya, 2016; de Gois da Silva et al., 2018; Fukumura et al., 2018; Papazian et al., 2018; Du et al., 2019; Luo et al., 2019; Upadhya et al., 2019; Datta et al., 2020; Lu et al., 2020). Additionally, they confer several therapeutic effects including attenuated neuronal death, boosted neurogenesis, repressed microglia and astrocyte reactivity, and mitigated neuroinflammation (Li et al., 2009; Costa-Ferro et al., 2010; Abdanipour et al., 2011). However, the neuropsychiatric component of epilepsy has still not been examined in animal models. Neuropsychiatric disorders often arise with the development of epilepsy. Neurogenesis in sectors of the brain that affect mood may ameliorate depression and other neuropsychiatric illnesses (Valenzuela et al., 2007). While stem cell transplantation may be a viable option for treatment of epilepsy, the use of cell therapy for treating both the neurologic and neuropsychiatric characteristics of epilepsy needs to be further evaluated.

## Recent Preclinical and Clinical Evidence Supporting the Safety and Efficacy of Stem Cell Therapy Against the Neurological Component of Epilepsy

### Pathophysiologic Processes of Epilepsy that Illuminate Therapeutic Targets for Stem Cell-Based Treatments

As numerous studies indicate, epilepsy’s pathology is intricate and encompasses a wide-range of mechanisms, several of which may serve as therapeutic targets for stem-cell based remedies. Indeed, irregular neuronal pathways, potentially engendered by the dysregulated construction of the neuronal network, has been associated with epilepsy (Homan et al., 2018; Schuster et al., 2019; Ahamad et al., 2020). Impaired dentate granule cell (DGC) development from NSCs may spur warped hippocampal activity, neuroplasticity, and formation of mature neurons, which would lead to hippocampal-related disorders like mesial temporal lobe epilepsy (MTLE) (Ahamad et al., 2020). It has been indicated that FHL2 protein is involved with dentate gyrus metabolism and neurogenesis (Ahamad et al., 2020). Findings suggested that FHL2 overexpression in DGCs induce heightened dendritic sprouting; whereas, FHL2 repression bolsters the length of dendrites (Ahamad et al., 2020). Moreover, understanding the mechanisms behind hippocampal deficits that manifest in MTLE is key to formulating cell-based therapeutic strategies to mitigate these impairments. Additionally, PCDH19-Girls Clustering Epilepsy (PCDH19-GCE) may also arise due to impaired neuronal development. PCDH19-GCE is generated by a mutation in the PCDH19 gene found on the X-chromosome (Homan et al., 2018). PCDH19 demonstrates elevated expression in human neural stem and progenitor cells (NSPCs). *In vitro*, PCDH19 deletion upregulated neurogenesis of mice NSPCs (Homan et al., 2018). Heightened neurogenesis was also found in human NSPCs lacking the PCDH19 gene (Homan et al., 2018). Moreover, these findings suggest that the heterochrony of neuron development is altered with the PCDH19 mutation, which may spur irregularities in the genesis of the neuronal network, resulting in the emergence of epilepsy (Homan et al., 2018). Regarding Dravet Syndrome (DS), iPSC GABA cells from DS patients displayed alterations in sodium current stimulation, aberrant reactivity to oxidative injury, and defective genes coding for chromatin formation, advancement of mitosis, and excitability (Schuster et al., 2019). Moreover, these findings suggest that GABAergic neurons generate irregular pathways in DS and elucidates a potential therapeutic strategy for regenerative treatment, replacing defective GABAergic neurons with healthy cells.

Novel research points to the link between epilepsy and aberrant calcium ion activity (Völkening et al., 2018; Xu et al., 2018; Avazzadeh et al., 2019). CaV1.2 and CaV1.3 are prominent L-type voltage-gated calcium channels (LTCCs) in the CNS and are coded for by Cacna1d (Völkening et al., 2018). LTCCs like CaV1.2 and CaV1.3 are involved in modulating the firing of neurons and stimulating Ca2+ dependent signalling, as well as regulating gene expression. Neuronal hyperexcitability that manifests in epilepsy may be spurred by LTCC impairment (Xu et al., 2018). Cav1.2 dysfunction has been shown to hinder hippocampal neurogenesis in type-1 astrocyte-like stem cells from the adult dentate gyrus (Xu et al., 2018). To investigate the role of CaV1.3, hippocampal NPCs were isolated from Cacna1d-deficient mice and examined *in vitro*. The lack of Cacna1d expression upregulated astrogenesis but suppressed neurogenesis. *In vivo*, NPCs in the dentate gyrus demonstrated weakened neuronal differentiation and proliferation (Xu et al., 2018). Moreover, administration of NPSs expressing normal Cacna1d may prove to be therapeutically potent in ameliorating the detrimental effects of mutated LTCCs associated with epilepsy. In addition to Cacna1d, altered expression of gene NRXN1 may also play a role in epilepsy (Avazzadeh et al., 2019). Omission of the NRXN1 gene is found in epilepsy, autism spectrum disorder (ASD), and schizophrenia (Avazzadeh et al., 2019). Compared to iPSCs derived from healthy individuals, iPSCs from ASD subjects displayed knockdown of NRXN1α, resulting in atypical calcium activity and ion transport/transporter function. Notably, voltage-gated calcium channels were drastically augmented (Avazzadeh et al., 2019). Moreover, aberrant calcium mechanisms induced by NRXN1 absence may be a potential target for stem cell therapy in epilepsy and its neuropsychiatric symptoms.

Additionally, epilepsy induces astrocyte malfunction, which plays a role in neuronal hyperexcitability. Hippocampal sclerosis (HS) induced by MTLE may involve the depletion of inter-astrocytic gap junction (GJ) coupling (Deshpande et al., 2020). MTLE was induced in mice with kaintate and HS developed. Heightened frequency of seizures occurred in the mice lacking GJ coupling and hyperexcitability of neurons increased significantly (Deshpande et al., 2020). Another study illustrated that BDNF and TrkB play a role in TLE-induced astrocyte impairment (Fernández-García et al., 2020). In lithium-pilocarpine mice, BDNF overexpression in astrocytes exacerbated TLE (Fernández-García et al., 2020). *In vitro*, abolishing astrocyte BDNF expression via knockdown of the BDNF gene attenuated the level of neuron firing and firing rate (Fernández-García et al., 2020). TrKB gene elimination in both hippocampal neurons and astrocytes resulted in neuroprotection in TLE mice, while the mice with astrocyte TrkB knockdown also demonstrated improved spatial learning (Fernández-García et al., 2020). Furthermore, astrocyte dysfunction in epilepsy may be amenable to cell-replacement therapy, as certain stem cells demonstrate the ability to differentiate into functioning astrocytes.

### Cell Migration and GABAergic Interneuron Differentiation

In epilepsy, stem cells have demonstrated the ability to replace GABAergic neurons that were depleted due to epilepsy, as they can differentiate into these neurons (Treiman, 2001). Epilepsy spurs the depletion of GABAergic neurons, leading to diminished inhibitory control in affected brain regions (Shetty and Upadhya, 2016). Therefore, GABAergic cell therapy aims to restore the lack of GABAergic neurons, thereby bolstering the inhibitory synaptic regulation in the epileptic cerebral regions (Shetty and Upadhya, 2016). Though grafts of engineered GABA producing cells have exhibited anti-seizure properties, these therapeutic effects are transient on account of low graft viability (Shetty and Upadhya, 2016). In preclinical studies, the efficacy of precursor cells harvested from the lateral ganglionic eminence and the medial ganglionic eminence (MGE) in the treatment of epilepsy has been investigated (Shetty and Upadhya, 2016). Notably, the combination of embryonic stem cells derived from the lateral ganglionic eminence, fibroblast growth factor 2, and a caspase inhibitor demonstrated significant curative potential, as the number of spontaneous recurrent seizures (SRS) drastically decreased (Shetty and Upadhya, 2016). Evidence indicates that MGE progenitor cells display great migratory ability, as they move to areas adjacent to the initial graft location (Shetty and Upadhya, 2016). In animal models, grafts of bilateral hippocampal precursor cells resulted in diminished seizure frequency (Hattiangady et al., 2008). The graft attenuated anomalous mossy fiber sprouting and stimulated GABAergic interneurons, which in turn rehabilitated the impaired neuronal circuitry in the epileptic brain region (Shetty and Upadhya, 2016). By upregulating the number of GABAergic interneurons, the hippocampal precursors augmented inhibitory control and as a result, injury in affected brain regions improved (Hattiangady et al., 2008). Interestingly, the inhibitory effect induced by stem cell transplantation may be due to the crosstalk among pyramidal neurons and recently developed interneurons (Hattiangady et al., 2008). In order to further elucidate the safety and efficacy of GABAergic cell therapy in the clinical context of epilepsy, the chronic repercussions of cell grafts, the effectiveness of this therapy against drug-resistant epilepsy, and this treatment’s utility in ameliorating the neuropsychiatric component of epilepsy should be investigated (Shetty and Upadhya, 2016).

NSCs display significant differentiative, migratory, and survival capacity, making them robust contenders for stem cell-based therapeutics against epilepsy. Preclinically, NSC transplantation has been shown to maintain normal levels of GABAergic inhibitory neurons and attenuate aberrant mossy fiber growth, both of which contribute to hippocampal excitability associated with epilepsy (Scheibel et al., 1974; Rao et al., 2006). NSC transplantation demonstrates significant neuroprotective potential in combatting epileptic seizures, as NSC grafting repressed anomalous neuronal firing, upregulated the release of neurotrophic factors, and diminished atypical mossy fiber growth (Scheibel et al., 1974; Rao et al., 2006). The transplantation of NSCs culminates in the production of new GABAergic interneurons and additional astrocytes (Shetty and Upadhya, 2016). Importantly, glial-derived neurotrophic factor (GDNF) expression is upregulated with the production of new astrocytes, and GDNF has been shown to bear anticonvulsant abilities (Waldau et al., 2010). NSC transplantation aids in lowering the amount of seizures and shortening the seizure duration (Waldau et al., 2010). The therapeutic efficacy of NSCs can also be observed in the pilocarpine induced TLE model. Notably, only 13% of pilocarpine rats continued experiencing SRS following β-galactosidase–encoded human NSC transplantation (Scheibel et al., 1974). In addition, the NSCs migrated to the CA1 and CA3 regions of the hippocampus, but only a few differentiated into mature neurons, expressing NeuN (Scheibel et al., 1974). A number of the NSCs were able to differentiate into GABAergic neurons, as depicted by the expression of specific markers (Scheibel et al., 1974). In the dentate gyrus of a TLE mice model, NSCs effectively evolved into GABAergic interneurons (Maisano et al., 2012). Following the graft transplantation of MGE-derived NSCs, rats exhibited a 43% reduction in SRS occurrence, and the cells that evolved from the NSCs migrated to the hippocampus, specifically to the CA3 and CA1 regions (Shetty, 2014). At three months post-grafting, the NSC graft displayed a survival rate of 30% and a high percentage of cells that survived gave rise to GABAergic neurons (Shetty, 2014).

A recent study examined whether neural precursor cell (NPS) grafts extracted from a range of species (e.g. rat, human, pig) could engender anticonvulsant outcomes in adult rats (Backofen-Wehrhahn et al., 2018). NPSs were isolated from two different sources: MGE and ventral mesencephalon (VM) (Backofen-Wehrhahn et al., 2018). Notably, in adult rats, the NPSs from both sources demonstrated great migratory ability, differentiated into inhibitory interneurons, and displayed significant graft viability, as the grafts survived for 4 months (Backofen-Wehrhahn et al., 2018). NPCs that were isolated from the VM of humans and pigs demonstrated anticonvulsant abilities when delivered to the subthalamic nucleus (STN) but not the substantia nigra pars reticulata (SNr), indicating that NPCs’ therapeutic potency may be dependent on injection site (Backofen-Wehrhahn et al., 2018). On the other hand, NPCs extracted from MGE failed to induce anticonvulsant effects in both the STN and SNr (Backofen-Wehrhahn et al., 2018). Since the findings indicate that NPCs harvested from different sources generate varying results, further investigation is warranted in order to delineate the most effective type of NPC in epileptic treatment. In another study, NSCs were extracted from new-born rats and evolved into GABAergic neurons *in vitro* (Backofen-Wehrhahn et al., 2018). Then NSCs and GABAergic neurons were transplanted into the hippocampus of pharmacoresistant epileptic rats treated with pilocarpine (Backofen-Wehrhahn et al., 2018). Both the NSC and GABAergic rats demonstrated a reduction in the recurrence of electroencephalography; however, the rats treated with the GABAergic neurons displayed the greatest cell migration to the hippocampus (Xu et al., 2019). Moreover, NSC-derived GABAergic neuron intrahippocampal transplantation exhibits substantial therapeutic efficacy in generating GABA-related inhibitory effects, thereby repressing SRS (Xu et al., 2019). In addition, PET imaging was employed to assess dynamic metabolic alterations in TLE rats following NSC and human GABA progenitor cell (GPCs) administration (Du et al., 2019). Glucose metabolism showed slight amelioration with NSCs but was exacerbated in the GPC and control groups (Du et al., 2019). Both NSCs and GPCs quelled seizures and demonstrated great viability, migratory capabilities, and differentiative potency (Du et al., 2019).

Interneuron precursor cells derived from various stem cell sources have demonstrated significant curative potential in epilepsy due to their robust homing capabilities. *In vitro*, medial and caudal ganglionic eminences (MGE/CGE) like progenitor cells derived from human pluripotent stem cells (hPSCs) evolved into mature striatal interneurons, as the cells displayed cortical and striatal interneuron mRNAs and proteins (Noakes et al., 2019). When the progenitor cells were delivered to the striatum of neonatal rats, the cells developed into subclasses of striatal interneurons and effectively migrated to the hippocampus (Noakes et al., 2019). In addition, human periventricular endothelial cells substantially expedited migration of human interneurons in co-culture and improved interneuron graft efficacy *in vivo* by bolstering homing capacity (Datta et al., 2020) Consequently, behavioral deficits were ameliorated in the psychiatric disorder animal model (Datta et al., 2020). Furthermore, interneuron grafts may alleviate the neuropsychiatric aspect of epilepsy by mitigating GABA-ergic neuron deficiency. Although interneuron precursors derived from hPSCs display therapeutic promise, hPSC differentiation into these precursor cells occurs at a slow rate. Therefore, recent studies have focused on finding ways to accelerate hPSC differentiation. For instance, hPSC differentiation into GABA interneurons (GINs) can be expedited with a combination of smoothened agonist (SAG), Forskolin, and azidothymidine (AZT) *in vitro* (Shen et al., 2020).

Notably, interneuron precursor cells have showcased great ability to ameliorate various pathological manifestations of epilepsy. When hiPSC-derived MEG-like interneuron precursor cells were transplanted into the hippocampus of an SE model, the cells effectively moved to the hippocampus and evolved into mature inhibitory interneurons, releasing a multitude of different neuropeptides (Upadhya et al., 2019). Importantly, the graft demonstrated substantial viability post SE (Upadhya et al., 2019). The grafted cells ameliorated SRS, along with cognitive, mood, and memory deficits that manifest in TLE’s chronic stage (Upadhya et al., 2019). The hiPSC-MGE cells also alleviated interneuron death, anomalous mossy fiber sprouting in the dentate gyrus, and aberrant neurogenesis, as well as assimilating well into synaptic networks (Upadhya et al., 2019). Of note, the administration of a drug that repressed hiPSC-MGEs significantly attenuated the graft’s therapeutic impact in inhibiting seizures (Upadhya et al., 2019). In another investigation, interneuron progenitors harvested from the embryonic MGE were transplanted into APP/PS1 mice, a model of Alzheimer’s disease (Lu et al., 2020). The progenitor cells displayed great viability and migratory capacity, and effectively evolved into GABAergic interneurons (Lu et al., 2020). The transplanted cells ameliorated dysfunctional synaptic plasticity in the hippocampus and attenuated hyperexcitability of neurons, thereby improving cognitive function (Lu et al., 2020). Moreover, interneuron progenitors may be equally beneficial in repressing hyperexcitability that manifests in epilepsy.

### Astrocyte Differentiation

In addition to GABAergic neuron differentiation, stem cells demonstrate the ability to evolve into astrocytes, which bears therapeutic potency in epilepsy, as astrogliosis is a critical feature of epileptic pathology. Recent evidence indicates that impaired astrocyte function is a critical component of epileptogenesis (Boison and Steinhäuser, 2018). Astrocytes play a crucial role in maintaining energy homeostasis of neurons (Boison and Steinhäuser, 2018) by generating the secretion of glutamate, ATP and d-serine in the synapse. Importantly, astrocytes maintain appropriate levels of glutamate and regulate glucose metabolism (Boison and Steinhäuser, 2018). Astrocytes function in inducing the sequestration of gliotransmitters like glutamate, and if neurotransmitter uptake is dysregulated, hyperexcitability of neuronal circuitry can arise (Boison and Steinhäuser, 2018). In mice with impaired astrocytes, the sequestration of potassium ions and glutamate was hindered, culminating in greater frequency of seizures and astrocyte swelling (Boison and Steinhäuser, 2018). The regulation of K+ levels by astrocytes is warped in epilepsy on account of heightened excitability of neurons (Wang F. et al., 2020). As K+ ions increase significantly in the extracellular space, a seizure is often spurred. Astrogliosis during an epileptic incident attenuates the entry of K+ into the cell in an active or passive manner (Wang F. et al., 2020). In both patients with Alzheimer’s disease (AD) and mice models of AD, increased phosphorylated tau has been found in GABAergic interneurons of the dentate gyrus (Zheng et al., 2020). Heightened phosphorylated tau spurred heightened astrogliosis, which led to reduced GABA levels and neuronal hyperexcitation (Zheng et al., 2020), as observed in epilepsy. Moreover, restoring proper astrocyte function via stem cell therapy may serve as an effective therapeutic strategy against epilepsy.

Preclinical studies have investigated the capacity of stem cells to replace impaired astrocytes and therefore, restore normal excitatory levels of neurons in epilepsy. Remarkably, MSCs demonstrate significant capacity to alleviate epilepsy-induced alterations in astrocytes. Changes in astrocytes spurred by inflammation associated with epilepsy may be amenable to mesenchymal stem cell-derived exosome (MSC-Exo) treatment (Xian et al., 2019). *In vitro*, MSC-Exo was administered to a culture of hippocampal astrocytes conditioned with lipopolysaccharide (LPS) and these cells were then transplanted into SE mice (Xian et al., 2019). In both *in vitro* and *in vivo*, the astrocytes took in the exosomes, resulting in mitigated astrogliosis and inflammation (Xian et al., 2019). *In vitro*, atypical calcium dynamics and impairments in mitochondrial function were alleviated by MSC-Exo (Xian et al., 2019). NSCs also display great potential in differentiating into functioning astrocytes (Byun et al., 2020). *In vitro*, NSCs produced from human embryonic stem cells (hESCs) were able to differentiate into astrocytes at an 80% capacity (Byun et al., 2020). On account of astrocytes' anticonvulsant abilities, NSC differentiation into healthy astrocytes bears significant therapeutic promise is cell-based epilepsy treatment (Waldau et al., 2010). In rats treated with KA, adult SVZ-derived NSCs were transplanted into the hippocampus, resulting in a drastic attenuation of aberrant electroencephalography spiking two weeks following NSC delivery (Jing et al., 2009). Remarkably, NSCs differentiated into astrocytes, as indicated by glial fibrillary acidic protein expression, and mature neurons, as neuronal nuclei (NeuN) were expressed (Jing et al., 2009). Another investigation explored the delivery of GDNF to the hippocampus of rats with epilepsy (Paolone et al., 2019) via an implantable cell encapsulation mechanism. The source of GDNF was human colonel ARPE-19 cells, which provided continuous release of GDNF. Following treatment, frequency of seizures was decreased by 93% over a period of 3 months (Paolone et al., 2019) in the pilocarpine model. GDNF treatment rehabilitated astrocytosis and neurodegeneration in the hippocampus, as well as the depletion of GABAergic neurons (Paolone et al., 2019).

### Neuroprotection

Notably, stem cells display neuroprotective capacity in epilepsy due to their ability to suppress oxidative injury and glutamate excitotoxicity. Overexpression of glutamate receptors generates the rapid entry of cations, impairment of mitochondria, and the accumulation of reactive oxygen species (ROS), all of which contribute to the neurotoxicity and eventual cell death (Armada-Moreira et al., 2020). An abundance of recent evidence points to the therapeutic relevance of MSCs in repressing epileptogenesis. In a lithium-pilocarpine injection rat model, MSCs were systematically infused and resulted in MSC build up in the hippocampus, safeguarding GAD67+ and NeuN + neurons (Fukumura et al., 2018). MSC treatment hindered epileptogenesis, ameliorated neurological deficits, and attenuated anomalous mossy fiber sprouting in the hippocampus (Fukumura et al., 2018). Importantly, MSC transplantation has been shown to shield neurons from glutamate excitotoxicity associated with epilepsy in both vitro and *in vivo* (Papazian et al., 2018). MSC-induced neuroprotection can be linked to diminished expression of NMDA glutamate receptor (NMDAR), attenuated Ca2+ activity in neurons spurred by glutamate, and upregulated expression of genes correlated with stem cells (Papazian et al., 2018). Bone marrow mesenchymal stem cells conditioned medium (MSC CM) engendered the phosphatidylinositide 3-kinase/protein kinase B (PI3-K/Akt) pathway in neurons from the rat hippocampus in culture, thereby inhibiting apoptosis (Papazian et al., 2018). *In vitro*, MSC CM was shown to impart neuroprotection by suppressing the expression of the GluR1 α-amino-3-hydroxy-5-methyl-4-isoxazolepropionic acid (AMPA) receptors (AMPAR) subunit, which in turn diminished he release of glutamate (Papazian et al., 2018). In cultured cortical neurons from mice, MSCs inhibited NR1 and NR2A expression, which are N-Methyl-d-aspartic acid (NMDA) receptor (NMDAR) subunits that are associated with glutamate signalling (Papazian et al., 2018), and consequently, glutamate excitotoxicity was attenuated (Papazian et al., 2018).

NSCs can effectively combat oxidative stress and glutamate excitotoxicity associated with epilepsy. NSCs were intravenously administered to epileptic rats treated with pilocarpine, pentylenetetrazole, and picrotoxin (de Gois da Silva et al., 2018). When compared to the control, rats with NSCs demonstrated diminished glutathione, superoxide dismutase, and catalase levels (de Gois da Silva et al., 2018). Moreover, NSCs show therapeutic promise, as they bear antioxidant capabilities that can alleviate epilepsy-induced oxidative injury (de Gois da Silva et al., 2018). Regarding glutamate excitotoxicity, traumatic brain injury (TBI) rats received autologous NSCs extracted from the cortex of these neonatal rats (Luo et al., 2019). Hippocampal neuron viability in the CA1 region was significantly greater in the NSC group than the control (Luo et al., 2019). In addition, the NSCs attenuated excitotoxicity by repressing Glu secretion and safeguarding GABA-related inhibition in the hippocampus, thereby ameliorating cognitive defects in these rats (Luo et al., 2019). Moreover, the ability of NSCs to hinder glutamate excitotoxicity and preserve inhibitory control indicate its therapeutic efficacy in epilepsy (Luo et al., 2019). In addition, glutamatergic excitation, prevalent in the dentate gyrus of those afflicted with epilepsy, may be alleviated by NSC-derived GABAergic neurons, as these neurons express neuropeptide Y, which weakens glutamatergic excitation (Patrylo et al., 1999). Findings suggest that NSCs release stem cell factors in conjunction with higher concentrations of c-Kit, which serves as the ligand for the stem cell factors (Yasuhara et al., 2006). These stem cell factors may play a role in the mechanisms behind NSC-induced amelioration of epileptic symptoms.

### Anomalous Neurogenesis

Stem cell transplantation can ameliorate neurodegeneration that arises in the chronic stage of epilepsy. Indeed, epileptic seizures may generate aberrant neurogenesis, which further accelerates epileptogenesis and exacerbates cognitive function (Martín-Suárez et al., 2020). Hippocampal neurogenesis proceeds when NSCs proliferate at significant levels in the dentate gyrus (Martín-Suárez et al., 2020). Since proper hippocampal neurogenesis is critical for memory, learning, and response to stress, warped neurogenesis in the hippocampus may serve as a therapeutic target for stem-cell based treatment of both the neurological and neuropsychiatric components of epilepsy (Martín-Suárez et al., 2020). Patients with epilepsy demonstrate substantial detrimental changes in hippocampal neurogenesis (Martín-Suárez et al., 2020). Preclinically, TLE-induced seizures spur the formation of reactive-NSCs, which in turn generates anomalous neuronal migration, morphology, and functionality (Martín-Suárez et al., 2020). In Dravet syndrome mice, an appreciable level of rogue neurogenesis was observed (Martín-Suárez et al., 2020). Additionally, the mitigation of anomalous neurogenesis spurred by seizures during the clinically relevant window resulted in the repression of SRS in models of MTLE (Varma et al., 2019). Though neurogenesis during the acute phase of epilepsy is detrimental, neurogenesis in the chronic stage is vital for rehabilitating neurodegeneration (Andres-Mach et al., 2011; Zhong et al., 2016). Moreover, stimulating normal hippocampal neurogenesis via stem cell therapy may be effective in inhibiting recurrent seizures in the chronic phase of epilepsy. Various stem cells have been explored as potential therapeutic agents in treating neurodegeneration spurred by epilepsy, such as human bone marrow mesenchymal stem cells (hBM-MSCs), NSCs, and endothelial progenitor cells (EPCs) (Ali et al., 2019; Dever et al., 2019; Kodali et al., 2019). Extracellular vesicles (EVs) derived from human bone marrow mesenchymal stem cells (hMSCs) were intranasally delivered to SE rats (Kodali et al., 2019). Compared to the control, a greater number of EVs integrated themselves into hippocampal neurons in the SE group, particularly in the CA1 region and entorhinal cortex, which are areas that normally suffer from neurodegeneration following SE (Kodali et al., 2019). Moreover, hMSC-derived EVs demonstrate ability to migrate to injured regions and may alleviate neurodegeneration due to their neuroprotective capabilities (Kodali et al., 2019). Regarding neural stem cells, designer NSCs were transplanted into oligodendrocyte mutant shiverer-immunodeficient mice. The cells homed to injured sites and developed into mature neurons, astrocytes and oligodendrocytes (Dever et al., 2019). Furthermore, genetically modified NSCs exhibit significant potential in alleviating neurodegeneration associated with epilepsy, as well as generating healthy astrocytes that bear anticonvulsant capabilities (Waldau et al., 2010). In another study, endothelial progenitor cells (EPCs) were intravenously delivered to rats treated with pentylenetetrazole (PTZ) (Ali et al., 2019). The EPCs effectively migrated to the hippocampus, alleviated neurological deficits, and upregulated BDNF expression (Ali et al., 2019). The cells also improved memory and motor function, as well as mitigating aberrations in neurotransmitter mechanisms and increasing expression of genes associated with autophagy (Ali et al., 2019). EPCs’ ability to stimulate autophagy suggests that EPC-based therapy may be beneficial in ameliorating epilepsy-induced neurodegeneration.

### Stem Cell Modifications and Their Value as Therapeutics for Epilepsy

Recent studies have explored the efficacy of stem cell therapy combined with other treatments, as well as genetically engineered stem cells in abating epilepsy. One such investigation examined whether Rhynchophylline (Rhy) may enhance stem cell metabolism, bolstering its therapeutic efficacy in neurodegenerative disorders like epilepsy (Kaneko et al., 2020). When Rhy was administered to bone marrow human mesenchymal stromal cells (SM-hMSCs), Rhy altered mitochondrial activity, fibroblast growth factor (FGFβ) and BDNF concentrations (Kaneko et al., 2020). Rhy also influenced metabolism by modifying oxytocin receptors and ATP levels (Kaneko et al., 2020). Rhy amended expression of genes associated with proliferation and differentiation in BM-hMSCs (Kaneko et al., 2020). Moreover, Rhy in conjunction with stem cell therapy may result in bolstered stem cell metabolism, generating even greater therapeutic benefits (Kaneko et al., 2020). Additionally, genetically modifying stem cells to increase their potency has become a significant area of focus and various methods of genetically engineering stem cells in the context of epilepsy are being explored. For instance, human adult neural stem/progenitor cells (NS/PSCs) were extracted from medically intractable epileptic patients during surgery (Abdolahi et al., 2020). These cells were then designed to express a green fluorescent protein (GFP) and the gene was delivered via lentivirus (Abdolahi et al., 2020). *In vitro*, approximately 80% of the NS/PCs were transduced by the lentivirus vector and GFP expression was apparent from 3 days following transduction up to at least four weeks (Abdolahi et al., 2020). Moreover, the lentivirus is an effective means of genetically engineering NS/PCs (Abdolahi et al., 2020). Interestingly, fibroblast growth factor 21 (FGF-21) has been shown to shield neurons from glutamate excitotoxicity (Linares et al., 2020). FGF-21 overexpression in BM-MSCs may boost its therapeutic potential by safeguarding the stem cells from apoptosis (Linares et al., 2020). BM-MSCs were genetically modified to overexpress FGF-21 through lentivirus transduction. Heightened expression of FGF-21 significantly attenuated hydrogen peroxide and TNF-α-induced caspase pathways (Linares et al., 2020). Indeed, BDNF plays an important role in regulating synaptic activity and GABAergic neuron viability (Kim HS. et al., 2020). Human neural stem cells overexpressing BDNF (HB1.F3. BDNF) were transplanted into a Huntington’s disease rat model (Kim HS. et al., 2020). The cells moved to the QA-lesioned striatum, differentiated into GABAergic neurons, and integrated effectively into the host’s cerebral network (Kim HS. et al., 2020). In addition, neuroinflammation was substantially alleviated (Kim HS. et al., 2020). Moreover, human NSCs designed to overexpress BDNF may exhibit therapeutic potential in epilepsy due to their migratory, differentiative, and anti-inflammatory abilities.

### Stem Cell Clinical Trials in Epilepsy

Clinically, the safety and efficacy of stem cell therapy in epileptic patients have been investigated. Adults suffering from MTLE received an intra-arterial autologous bone marrow mononuclear cell (BMMC) injection and were assessed over 6 months (DaCosta et al., 2018). At the end of the observation period, 40% of the subjects no longer had seizures. In addition, memory scores escalated substantially. Importantly, the stem cell transplantation was safe, exhibiting no adverse events (DaCosta et al., 2018). In another clinical trial, autologous adipose-derived regenerative cells (ADRCs) were intrathecally administered via liposuction to autoimmune refractory epileptic patients (Szczepanik et al., 2020). They received treatment three times for every three months (Szczepanik et al., 2020). The patients demonstrated cognitive recovery, as observed in ameliorated academic, social, and motor performance. One subject became completely seizure free, but other patients showed less seizure mitigation or no improvement at all (Szczepanik et al., 2020). Despite ADRCs apparent safety, its efficacy must be further explored in the context of epilepsy (Szczepanik et al., 2020). In addition, a clinical trial utilizing pediatric patients investigated the effectiveness of autologous bone marrow stem cell transplantation in ameliorating drug-resistant epilepsy (DRE) (Milczarek et al., 2018). Every three months, the subjects received an intrathecal or intravenous dose of bone marrow nucleated cells (BMNCs) in conjunction with intrathecal bone marrow mesenchymal stem cells (BMMSCs) (Milczarek et al., 2018). After two years, the treatment proved to be safe with no adverse incidents and demonstrated positive results, ameliorating neurological function and lowering frequency of epileptic seizures (Milczarek et al., 2018). Indeed, recent clinical trials have displayed encouraging findings; however, further preclinical investigation into the safety and efficacy of stem cell therapy in ameliorating the neurological and neuropsychiatric aspects of epilepsy is warranted to ascertain optimal dosage, timing, and delivery method.

## The Efficacy of Stem Cells in Alleviating the Neuropsychiatric Comorbidities of Epilepsy

### Regulating Adenosine Homeostasis

In addition to addressing the neurological deficits engendered by epilepsy, stem cells also show potential in ameliorating epilepsy’s neuropsychiatric aspect. Epilepsy, along with depression and schizophrenia, have exhibited mutated adenosine homeostasis, which may serve as a therapeutic target in cell-based therapy (Poppe et al., 2018). Heightened energy metabolism in a epileptic event manifests in a significant rise of adenosine levels (Boison and Steinhäuser, 2018). Indeed, astrocytes and adenosine kinase (ADK) play an important role in regulating adenosine homeostasis in the brain (Boison and Steinhäuser, 2018). Previous investigations have indicated that ADK bears an important function in cerebral development postnatally. A study found that knockdown of the Adk gene resulted in altered neuroplasticity, which spurred seizures, as well as imparired memory and learning (Gebril et al., 2020). Mutated ADK expression during brain development is correlated with anomalous adenosine homeostasis, which has been linked to neuropsychiatric maladies like autism and schizophrenia (Gebril et al., 2020). *In vitro*, zinc finger nuclease (ZFN) was utilized to delete the ADK gene in neuroepithelial stem cells (NES) extracted from human embryonic stem cells. A drastic increase in adenosine secretion was observed in ADK lacking It-NES cells than the control (Poppe et al., 2018). Furthermore, these cells may serve as effective adenosine carriers and may be therapeutically potent in ameliorating epilepsy-induced alterations in adenosine levels (Poppe et al., 2018), which would address both the neurological and neuropsychiatric aspects of epilepsy. In addition, astrogliosis in intractable epilepsy spurs boosted expression of ADK, leading to adenosine scarcity (Estiri et al., 2018). A lentivirus vector carrying anti-ADK miR-shRNA abolished ADK expression at a 95% capacity in Wharton's jelly mesenchymal stem cells (hWJMSCs) and the astrocytoma cell line (Estiri et al., 2018). Moreover, genetically modified WJMSCs may be effective in drastically improving adenosine levels in epilepsy and therefore, demonstrates therapeutic potential in alleviating the neuropsychiatric comorbidities of epilepsy.

### The Role of the Amygdala in Epilepsy and Implications for Neuropsychiatric Comorbidities

Interestingly, targeted stem cell therapy to the amygdala may be a potent therapeutic strategy in epilepsy. Indeed, patients with drug resistant lesional and nonlesional TLE have frequently demonstrated amygdala enlargement (AE) (Peedicail et al., 2020). Out of 42 patients, 33 displayed AE and 11 of these patients also showed hippocampal enlargement (HE). In a case study with a TLE patient demonstrating neuropsychiatric complications (e.g. psychosis, depression, irritability), anterior temporal lobectomy and amygdalohippocampectomy resulted in a decrease in seizure frequency and the amelioration of the neuropsychiatric manifestations (Shahani and Cervenka, 2019). Moreover, amygdala injury is significant feature of epilepsy pathology, thereby indicating a potential treatment target for both neurological and neuropsychiatric symptoms. Notably, an *in vitro* investigation found that neurospheres derived from the adult human amygdala afflicted with intractable epilepsy differentiated into human neural stem/progenitor cells (hNS/PCs) and astrocytes (Ghasemi et al., 2018). With administration of SHH and retinoic acid, these cells were able to differentiate into motor neurons (Ghasemi et al., 2018). Moreover, the amygdala may serve as a source for autologous stem cell therapy in epilepsy and may demonstrate potential in treatment of the neuropsychiatric disorders associated with epilepsy like anxiety (Ghasemi et al., 2018). The activity of multipotent NSCs play a key role in hippocampal neurogenesis in the adult brain (Muro-García et al., 2019). MTLE was mirrored in mice that received kainic acid (KA) in the amygdala. The KA spurred the transformation of NSCs to React-NSCs, leading to aberrant neurogenesis. Indeed, evidence indicates that cognitive impairment, anxiety and depression in patients with MTLE may be due to anomalous neurogenesis induced by epilpetic seizures (Muro-García et al., 2019). The fact that KA injection into the amygdala showed similar effects to hippocampal delivery further suggests the role of the amygdala in epilepsy, as well as a potential mechanism for anxiety that accompanies epilepsy. Additionally, the amygdalae harvested from MTLE patients demonstrated neuronal death and gliosis (Jafarian et al., 2019). In epilteptic patients, the amygdalae demonstrated escalated neuronal impairment and apoptosis, along with reduced GABAergic receptor subunit expression (Jafarian et al., 2019). However, levels of NR1, NR2B, mGluR1α, GluR1, and GluR2, which are glutamate excitatory receptor subunits, were similar to the control group (Jafarian et al., 2019). On account of stem cells’ ability to differentiate into GABAergic neurons, targeted cell therapy in the amygdala may serve as an effective therapeutic strategy against epilepsy as well as its neuropsychiatric comorbidities.

### Neurotrophic Factors

Evidently, alterations in expression of neurotrophic factors, such as BDNF and GDNF, have been observed in epilepsy and neuropsychiatric disorders, and aberrant expression of these factors may be amenable to stem cell therapy. Indeed, *in vivo* studies have demonstrated that BDNF depletion spurs an anomalous response to stress and BDNF has been implicated in chronic stress disorders like PTSD (Notaras and van den Buuse, 2020). It has been shown that glucocorticoid pathways upregulate BDNF, resulting in intensified fear memory (Notaras and van den Buuse, 2020). Additionally, BDNF plays a key role in epilepsy pathology in that it is substantially downregulated in epilepsy models (Ali et al., 2019). BDNF is involved with differentiation of hippocampal neurons, as well as the transmission of neurotransmitters like dopamine, serotonin, and glutamate (Ali et al., 2019). Diminished BDNF expression in the hippocampus has been correlated with impaired learning and memory (Ali et al., 2019). Through PET imaging, pilocarpine-induced epilpetic rats showed thalamic hypometabolism, diminished [18F]FDG and upregulated septal [18F]MPPF, which have been linked to changes in behavior and anxiety (Di Liberto et al., 2018). These thalamic and septal changes were associated with decreased BDNF in blood serum (Di Liberto et al., 2018). Moreover, epilepsy-induced alterations in BDNF expression may play a role in engendering the neuropsychiatric comorbidities of epilepsy. In addition, endothelial progenitor cells (EPCs) were delivered to the hippocampus of PTZ-induced epilepsy rats (Ali et al., 2019), resulting in improved memory and motor capacity. EPCs ameliorated neuronal injury, upregulated BDNF expression, alleviated impaired neuronal circuitry, and elevated the expression of autophagy-related proteins, which was suppressed in epileptogenesis (Ali et al., 2019). By increasing BDNF levels, EPCs may mitigate the neurological complications of epilepsy, as well as neuropsychiatric comorbidities like anxiety, PTSD, and cognitive impairments. Additionally, rats treated with intrahippocampal KA received adipose-derived stem cells (ADSCs) (Wang et al., 2021). The ADSCs demonstrated significant differentiative capacity, as observed with the high levels of Tuj1, MAP2, NeuN, and PSD-95 expression in these cells (Wang et al., 2021). Importantly, the rats completed the Morris water maze task more effectively following ADSC treatment, indicating ameliorated learning and memory deficits spurred by epilepsy (Wang et al., 2021). The ADSCs also secreted neurotrophic factors like BDNF, NT3, and NT4, which may play a role in alleviating cognitive impairments accompanying epilepsy (Wang et al., 2021).

Of note, GDNF may also play a role in epilepsy-induced neuropsychiatric compilations. The cerebrospinal fluid (CSF) of patients with schizophrenia, bipolar disorder, and major depressive disorder was examined (Hidese et al., 2020). Concentrations of GDNF and CSF amyloid precursor protein (APP) were substantially reduced in schizophrenic patients (Hidese et al., 2020). APP and neural adhesion molecule 1 (NACAM-1) was significantly downregulated in BD patients (Hidese et al., 2020). Furthermore, utilizing stem cells to deliver these neurotrophic factors may be therapeutically potent in epilepsy. GDNF-releasing ARPE-19 cells were transplanted in quinolinic acid rats, a model of Huntington’s disease (Emerich et al., 2019). Treatment resulted in effective GDNF permeation throughout the rat striatum (Emerich et al., 2019). GDNF ameliorated behavioral impairments and functioned to maintain healthy striatal size (Emerich et al., 2019). In a similar study, GDNF delivery to the hippocampus via encapsulated GDNF-release cells resulted in decreased anxiety and alleviated seizure frequency, indicating that GDNF therapies may also address the neuropsychiatric comorbidities of epilepsy (Paolone et al., 2019).

### Glutamate Excitotoxicity

Glutamate excitotoxicity has been implicated in epilepsy, as well as in neuropsychiatric illnesses like schizophrenia and obsessive-compulsive disorder (OCD) (Karthik et al., 2020). Glutamate plays a critical role in regulating cell migration in the striatum. However, a stable concentration of glutamate must be maintained to prevent neuronal complications (Karthik et al., 2020). Glutamate is heavily involved with the cortico-striato-thalamo-cortical (CSTC) pathway, which may become aberrant in OCD (Karthik et al., 2020). OCD patients who were not taking medication demonstrated substantially greater concentrations of glutamate in CSF compared to the control group (Karthik et al., 2020). Among epilepsy patients, OCD is a frequent neuropsychiatric comorbidity that manifests, but it often goes untreated (Kim SJ. et al., 2020). 40.3% of 221 epileptic adults displayed an Obsessive-Compulsive Inventory-Revised (OCI-R) score of greater than or equal to 21, indicating that OCD is a common comorbidity of epilepsy, including severe epilepsy and TLE (Kim SJ. et al., 2020). Some AED treatments like lamotrigine may exacerbate OCD in patients. Therefore, exploring other therapeutics that effectively mitigate the neurological and neuropsychiatric components of epilepsy are critical for bettering the lives of epileptic patients. Stem cells demonstrate the capacity to alleviate glutamate excitotoxicity, thereby showing potential to reduce the frequency of seizures and treat OCD that may accompany epilepsy. Glutamate excitotoxicity has also manifested in the pathology of schizophrenia, as it spurs structural changes in cortical thickness (Shah et al., 2020). Imaging of the brain from ultra-treatment-resistant schizophrenia (UTRS) patients revealed significant thinning of the cortex (Shah et al., 2020). Schizophrenia, as a comorbidity of epilepsy, may be amenable to stem cell regenerative therapy. Lastly, fear related seizures, known as ictal fear (IF), arise in a certain class of MTLE patients on account of epilepsy-induced alterations in the defensive survival circuitry of the brain (Leal et al., 2020). These variations are thought to be correlated with the phosphorylation of Ser831 and Ser848, which are components of the GluA1 subunit (Leal et al., 2020). Compared to the control, MTLE patients with hippocampal sclerosis showed an 11% downregulation of Ser845 and Ser831 phosphorylation in the amygdala (Leal et al., 2020). By interacting with the glutamate receptors, stem cells may be able to correct the anomalous survival circuitry that manifests with IF epilepsy.

## Conclusion

In the current state, epilepsy impacts millions of people world-wide and its devastating cerebral effects can only be regulated with limited therapeutic options but not fully eliminated. While symptoms may be treated with the use of AEDs and surgical resection, the neuropsychiatric aspect of epilepsy is usually overlooked. Indeed, autologous stem cell transplantation has emerged as a potential therapeutic approach for epilepsy. The therapeutic potency of NSCs, MSCs, and interneuron precursors in epilepsy has been explored preclinically and in some clinical trials. These stem cells demonstrate the ability to ameliorate aberrant neuronal circuitry and restore depleted GABA-ergic inhibitory neurons, as well as exert neuroprotection against glutamate excitotoxicity and oxidative stress. Stem cell therapy may also effectively mitigate the neuropsychiatric comorbidities of epilepsy by maintaining adenosine homeostasis, targeting GABAergic neuron loss in the amygdala, releasing neurotrophic factors, and suppressing glutamate excitotoxicity. Nevertheless, further preclinical investigation into the safety and efficacy of stem cell therapy in clinically relevant models of epilepsy, incorporating both neurological and neuropsychiatric aspects is warranted. Before findings can be translated to clinical trials, optimal stem cell dosage, delivery method, and timing of transplantation must be established.
